# Prognostic value of the systemic immune-inflammation index in patients with breast cancer: a meta-analysis

**DOI:** 10.1186/s12935-020-01308-6

**Published:** 2020-06-09

**Authors:** Yantao Zhang, Yong Sun, Qiwen Zhang

**Affiliations:** Department of Two Gland Surgery, Jinan People’s Hospital Affiliated to Shandong First Medical University, Jinan, Shandong 271100 China

**Keywords:** Breast cancer, Meta-analysis, Systemic immune-inflammation index, Prognosis, Tumor microenvironment

## Abstract

**Background:**

Although previous studies have evaluated the prognostic role of the systemic immune-inflammation index (SII) in patients with breast cancer, the results were inconsistent. Therefore, in this context, we aimed to identify the prognostic and clinicopathological value of the SII in patients with breast cancer by performing a meta-analysis.

**Methods:**

A literature search was using PubMed, Web of Science, EMBASE, and Cochrane Library databases for relevant articles, from their inception to May 12, 2020. The prognostic value of the SII in breast cancer was assessed by pooling the hazard ratios (HRs) with 95% confidence intervals (CIs). The clinical outcomes included the overall survival (OS), disease-free survival (DFS), recurrence-free survival (RFS), and distant metastasis-free survival (DMFS). The methodological quality of all the included studies was evaluated using the Newcastle–Ottawa quality assessment scale. The odds ratios (ORs) with 95% CIs were combined to evaluate the correlation between the SII and clinicopathological characteristics of patients with breast cancer. Publication bias was evaluated using the Begg funnel plot and the Egger linear regression test. All statistical analyses were performed using Stata software, version 12.0 (Stata Corporation, College Station, TX, USA). A *p* value of < 0.05 was considered statistically significant.

**Results:**

Eight studies involving 2642 patients were included in the current meta-analysis. The combined data showed that patients with a high SII had worse OS (HR = 1.79, 95% CI 1.33–2.42, p < 0.001), poorer DFS/RFS (HR = 1.79, 95% CI 1.31–2.46, p < 0.001), and inferior DMFS (HR = 1.64, 95% CI 1.32–2.03, p < 0.001) than patients with a low SII. In addition, a high SII was correlated with the presence of lymph node metastasis (OR = 1.38, 95% CI 1.12–1.69, p = 0.002), higher T stage (OR = 1.49, 95% CI 1.17–1.89, p < 0.001), advanced TNM stage (OR = 1.37, 95% CI 1.07–1.77, p = 0.014), and higher histological grade (OR = 3.71, 95% CI 1.00–13.73, p = 0.049). However, there was no significant association between the SII and the pathological type (OR = 0.82, 95% CI 0.55–1.23, p = 0.345) or lymphatic invasion (OR = 1.30, 95% CI 0.82–2.08, p = 0.266).

**Conclusions:**

The results of our meta-analysis suggest that an elevated SII predicts poor survival outcomes and is associated with clinicopathological features that indicate tumor progression of breast cancer.

## Background

Breast cancer is the most common malignancy and the leading cause of cancer-related deaths in women worldwide [1]. In 2018, approximately 2,088,849 new cases of breast cancer and 626,679 deaths occurred worldwide [1]. Over the past several decades, the mortality due to breast cancer has decreased in Europe and in the United States because of early diagnosis and systemic treatments [2]. For patients with breast cancer with local and metastatic disease, the treatment approaches include surgery, radiotherapy, and systematic treatment with chemotherapy, endocrine therapy, and targeted therapy, or a combination of these [3]. However, the clinical outcomes of patients with breast cancer remain unsatisfactory owing to a lack of effective prognostic factors. Therefore, novel and reliable prognostic parameters need to be identified for designing personalized treatment regimens and for improving the survival of patients with breast cancer.

Tumor environment and inflammation play important roles in tumor development [4]. The components of the tumor microenvironment include the response cells, such as neutrophils, monocytes, lymphocytes, platelets, and cytokines. Several inflammatory cell parameters, including the neutrophil-lymphocyte ratio, platelet-lymphocyte ratio, C-reactive protein/albumin ratio, and systemic immune-inflammation index (SII), are derived using these meditators. The SII is an index that is calculated on the basis of the platelet, neutrophil, and lymphocyte counts. The SII has been used to evaluate the pretreatment balance between inflammatory factors and immune status of patients with cancer [5–8]. The SII is associated with the prognosis of patients with breast cancer, although the results are controversial [9–16]. Therefore, we performed the current meta-analysis to identify the prognostic impact of the SII in patients with breast cancer by aggregating all available data.

## Materials and methods

### Search strategy

The current meta-analysis was conducted according to the Preferred Reporting Items for Systematic Reviews and Meta-Analyses Statement [17]. A literature search was using PubMed, Web of Science, EMBASE, and Cochrane Library databases for relevant articles, from their inception to May 12, 2020. The following search terms were used: (systemic immune-inflammatory index or SII or systemic-immune-inflammation index or systemic immune-inflammation index) and (breast carcinoma or breast tumor or Breast Cancer or Breast Tumors or Cancer of Breast or Cancer of the Breast or Human Mammary Carcinoma or Mammary Carcinoma, Human or Mammary Neoplasm, Human or Mammary Neoplasms, Human or Neoplasms, Breast or Tumors, Breast or Breast Neoplasm or Breast Tumor or Cancer, Breast or Carcinoma, Human Mammary or Carcinomas, Human Mammary or Human Mammary Carcinomas or Human Mammary Neoplasm or Human Mammary Neoplasms or Mammary Carcinomas, Human or Neoplasm, Breast or Neoplasm, Human Mammary or Neoplasms, Human Mammary or Tumor, Breast). The references of the searched articles were also manually checked for additional relevant records. The language of publication was restricted to English. There were no restrictions on the study design (prospective or retrospective), location, or ethnicity. The current meta-analysis collected data from previously published studies; therefore, approval was not required from the ethical committee or medical institutional board.

### Inclusion and exclusion criteria

The inclusion criteria for eligible studies were as follows: (1) all patients were diagnosed with breast cancer; (2) studies reported the association between the SII and prognosis of patients with breast cancer; (3) a cutoff value was given for defining a high and a low SII; (4) the hazard ratios (HRs) with 95% confidence intervals (CIs) for survival outcomes were reported or sufficient data were given for calculating the HRs with 95% CIs. The following studies were excluded: (1) letters, reviews, and case reports; (2) duplicate studies; (3) studies with insufficient data; and (4) animal studies.

### Data extraction and quality assessment

Two independent investigators (Y.Z. and Y.S.) extracted the data from eligible studies by using a standardized form. Any disagreements were resolved via discussion with a third investigator (Q.Z.). The extracted information included the name of the first author, year of publication, country of study origin, study duration, molecular stratification of breast cancer, sample size, median age, clinical stage, ethnicity, treatment methods, SII cutoff value, method for cutoff determination, follow-up, survival outcomes, and HRs with 95% CIs. The clinical outcomes included the overall survival (OS), disease-free survival (DFS), recurrence-free survival (RFS), and distant metastasis-free survival (DMFS). The methodological quality of all the included studies was evaluated by using the Newcastle–Ottawa quality assessment scale (NOS) [18]. The NOS assesses the quality of the included studies by using a score of 0 to 9 points. Studies with a NOS score of ≥ 6 points were regarded as high-quality studies.

### Statistical analysis

The prognostic value of the SII in patients with breast cancer was assessed by pooling the HRs and 95% CIs. The odds ratios (ORs) with 95% CIs were combined to evaluate the correlation between the SII and clinicopathological characteristics of patients with breast cancer. The heterogeneity among studies was evaluated using the Cochran Q test [19] and the Higgins *I*^2^ statistics [20]. Significant heterogeneity was defined as p < 0.10 and/or *I*^2^ > 50%, and then, a random-effects model was applied for pooling the data. Otherwise, a fixed-effects model was applied. Subgroup analysis—stratified by the molecular stratification, cutoff value of the SII, method for cutoff determination, and treatment—was performed to explore the sources of heterogeneity. Publication bias was evaluated using the Begg funnel plot [21] and the Egger linear regression test [22]. All statistical analyses were performed using Stata software, version 12.0 (Stata Corporation, College Station, TX, USA). A p-value of < 0.05 was considered statistically significant.

## Results

### Search results and study characteristics

**Fig. 1:**
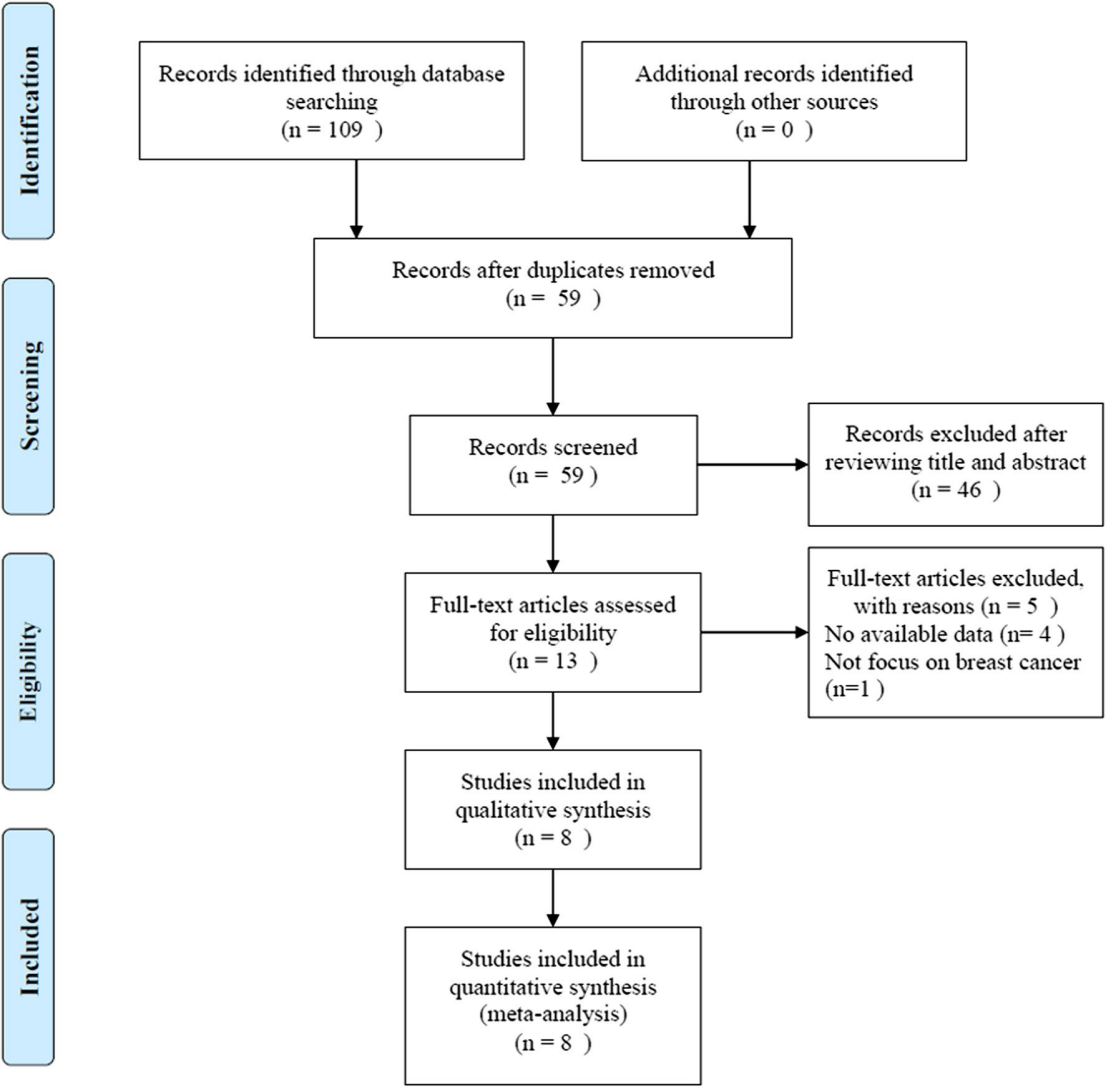
Schematic flow diagram for selection of included studies

A total of 109 studies were identified after the initial search of the databases, and then, 50 duplicate records were removed. After screening the title and/or the abstract, 46 studies were eliminated on the basis of the inclusion criteria. Then, 13 full-text articles were evaluated for eligibility [9–16, 23–27]. A total of 5 studies were removed owing to the following reasons: 4 studies [23–25, 27] did not provide sufficient data for the current meta-analysis, and 1 study [26] included patients with different cancers, rather than breast cancer only. Finally, 8 studies [9–16] involving 2642 patients were included in the current meta-analysis. A flowchart of the literature search is shown in Fig. 1. The general characteristics of the enrolled studies are summarized in Table 1. The included studies were published from 2019 to 2020 and were mainly conducted in 2 countries, including 1 in Italy [9] and 7 in China [10–16]. The total sample size was 2642 patients, ranging from 147 to 1026 patients. Seven studies reported the prognostic value of the SII considering OS [9, 11–16], 7 studies provided data on the association between the SII and DFS/RFS [10–16], and 3 studies reported the correlation between the SII and DMFS [11, 12, 16]. The cutoff values of the SII ranged from 422 to 836 in the included studies. Considering the quality assessment of the eligible studies, all the studies had a NOS score of ≥ 6 and the median value was 7, indicating that all the included studies were high-quality studies.

**Table 1:** Basic characteristics of all included studies

Author	Year	Country	Molecular stratification	Included period	Sample size	Age, years Median (range)	Clinical stage	Ethnicity	Treatment	Survival outcomes	Cut-off value (10^9)	Cut-off determination	Follow-up, months Median (range)	NOS score
De Giorgi	2019	Italy	Mixed	2004–2009	516	59	IV	Caucasian	No‐surgery	OS	836	ROC analysis	24	8
Li	2019	China	Luminal BC	2008–2013	161	58	I–III	Asian	No-surgery	DFS	518	ROC analysis	28.4 (1–79)	7
Liu	2019	China	TNBC	2000–2012	160	NA	I–III	Asian	Mixed	OS, DFS, DMFS	557	ROC analysis	61.7 (5.9–159.0)	6
Sun	2019	China	HER2+	2002–2012	155	NA	I–III	Asian	Mixed	OS, DFS, DMFS	578	Median value	57.6 (10.4–158.2)	6
Wang	2019	China	TNBC	2008–2016	215	NA	I–III	Asian	Mixed	OS, DFS	624	Median value	49.2 (4–105)	7
Chen	2020	China	Mixed	1999–2014	262	48 (27–73)	II–III	Asian	No-surgery	OS, DFS	602	ROC analysis	NA	8
Hua	2020	China	Mixed	2010–2012	1026	47 (22–87)	I–III	Asian	Mixed	OS, RFS, DMFS	601.7	ROC analysis	68.5 (0.9–87.5)	7
Jiang	2020	China	HER2+	2011–2015	147	NA	I–III	Asian	Mixed	OS, DFS	442	ROC analysis	42 (15–78)	7

*BC* breast cancer, *TNBC* triple-negative breast cancer, *HER2* human epidermal growth factor receptor-2, *OS* overall survival, *DFS* disease-free survival, *RFS* recurrence-free survival, *DMFS* distant metastasis-free survival, *ROC* receiver operating characteristics, *NA* not available, *NOS* Newcastle–Ottawa Scale

### Association between the SII and OS of patients with breast cancer

**Fig. 2:**
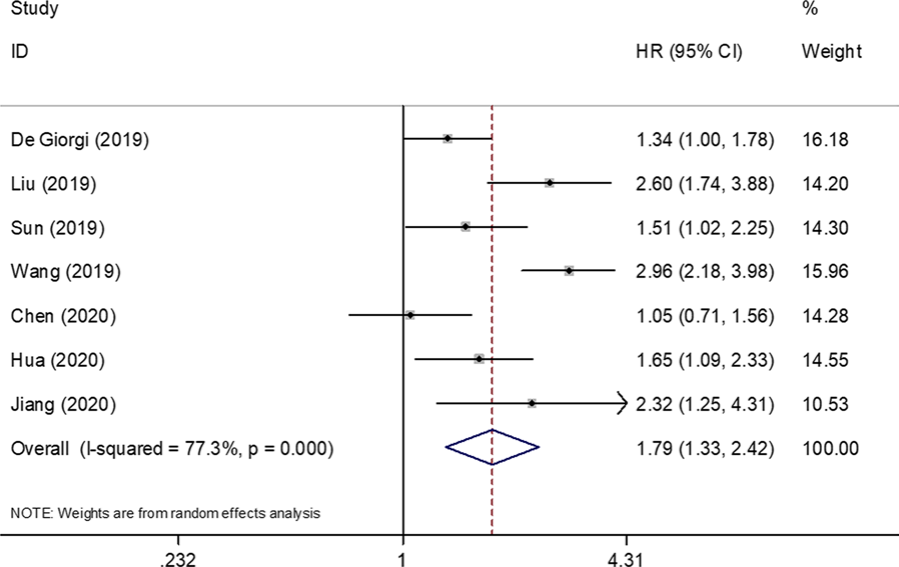
Forest plot of the correlation between SII and overall survival in patients with breast cancer

The data regarding the association between the SII and OS were available in 7 studies with 2481 patients [9, 11–16]. As shown in Fig. 2, the pooled HRs and 95% CIs revealed that patients with a high SII had worse OS (HR = 1.79, 95% CI 1.33–2.42, p < 0.001) than patients with a low SII. A random-effects model was used because of significant heterogeneity (*I*^2^ = 77.3%, p < 0.001; Fig. 2; Table 2). Subgroup analysis of OS was conducted on the basis of the molecular stratification, cutoff value of the SII, method for cutoff determination, and treatment. The subgroup analysis showed that an elevated SII was associated with poor OS of patients with triple-negative breast cancer (TNBC; HR = 2.82, 95% CI 2.22–3.59, p < 0.001), patients with breast cancer that was positive for human epidermal growth factor receptor-2 (HR = 1.71, 95% CI 1.23–2.39, p = 0.002), and patients with mixed molecular stratification (HR = 1.33, 95% CI 1.09–1.63, p = 0.005; Table 2). Considering the cut-off value of the SII, an SII cut-off value of ≤ 600 (HR = 2.03, 95% CI 1.57–2.62, p < 0.001) and an SII cut-off value of > 600 (HR = 1.63, 95% CI 1.04–2.56, p = 0.033) showed prognostic value for poor OS. Regarding the cut-off determination methods, both receiver operating characteristic curve analysis (HR = 1.65, 95% CI 1.20–2.26, p = 0.002) and the median value (HR = 2.14, 95% CI 1.11–4.14, p = 0.024) to determine the cut-off value were correlated with poor OS. Considering the treatment, a high SII showed a prognostic value for worse OS of patients receiving mixed treatments (HR = 2.15, 95% CI 1.61–2.86, p < 0.001; Table 2).

**Table 2:** Stratified analysis of pooled HR of breast cancer patients with SII on OS, DFS, RFS, and DMFS

Subgroup analysis	No. of studies	No. of patients	HR (95% CI)	p	Effects model	Heterogeneity	
						*I*^2^ (%)	p
OS							
Total	7	2481	1.79 (1.33–2.42)	< 0.001	Random	77.3	<0.001
Molecular stratification							
Mixed	3	1804	1.33 (1.09–1.63)	0.005	Fixed	21.9	0.278
TNBC	2	375	2.82 (2.22–3.59)	< 0.001	Fixed	0	0.612
HER2+	2	302	1.71 (1.23–2.39)	0.002	Fixed	23.9	0.252
Cut-off value of SII							
≤ 600	3	462	2.03 (1.57–2.62)	< 0.001	Fixed	47.3	0.150
> 600	4	2019	1.63 (1.04–2.56)	0.033	Random	85.9	< 0.001
Method for cut-off determination							
ROC analysis	5	2111	1.65 (1.20–2.26)	0.002	Random	68.7	0.012
Median value	2	370	2.14 (1.11–4.14)	0.024	Random	85.8	0.008
Treatment							
Mixed	5	1703	2.15 (1.61–2.86)	<0.001	Random	60.5	0.038
No-surgery	2	778	1.23 (0.98–1.56)	0.080	Fixed	0	0.330
DFS/RFS							
Total	7	2126	1.79 (1.31–2.46)	<0.001	Random	66.0	0.007
Molecular stratification							
Mixed	2	1288	1.34 (0.89–2.01)	0.160	Random	56.6	0.129
Luminal BC	1	161	6.04 (1.82–19.98)	0.003	–	–	–
TNBC	2	375	2.03 (1.06–3.88)	0.033	Random	81.0	0.022
HER2+	2	302	1.94 (0.83–4.56)	0.128	Random	52.0	0.149
Cut-off value of SII							
≤ 600	4	623	1.92 (1.20–3.08)	0.006	Random	57.2	0.072
> 600	3	1503	1.70 (1.01–2.88)	0.047	Random	81.1	0.005
Method for cut-off determination							
ROC analysis	5	1756	1.68 (1.14–2.47)	0.008	Random	60.7	0.038
Median value	2	370	2.02 (1.05–3.89)	0.035	Random	81.5	0.020
Treatment							
Mixed	5	1703	1.82 (1.36–2.42)	< 0.001	Random	52.5	0.078
No-surgery	2	423	2.31 (0.43–12.43)	0.328	Random	86.1	0.007
DMFS							
Total	3	1341	1.64 (1.32–2.03)	< 0.001	Fixed	0	0.590

### Association between the SII and DFS/RFS of patients with breast cancer

**Fig. 3:**
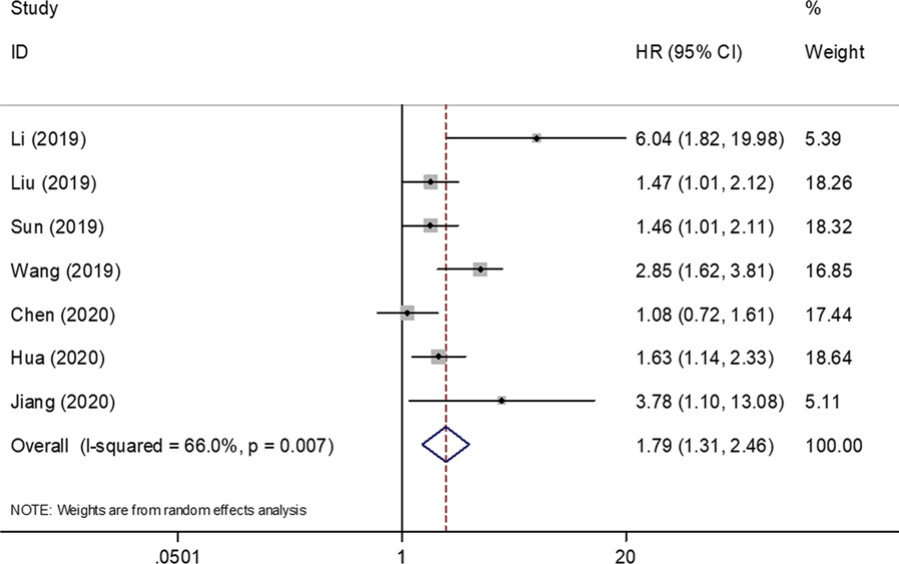
Forest plot of the correlation between SII and disease-free survival/recurrence-free survival in patients with breast cancer

A total of 7 studies consisting of 2126 patients [10–16] investigated the association between the SII and DFS/RFS. Owing to significant heterogeneity (*I*^2^ = 66.0%, p = 0.007), a random-effects model was applied (Fig. 3; Table 2). The combined data showed that an elevated SII was correlated with poor DFS/RFS of patients with breast cancer (HR = 1.79, 95% CI 1.31–2.46, p < 0.001; Fig. 3). Subgroup analysis stratified by molecular stratification showed that a high SII was associated with poor DFS/RFS of patients with luminal breast cancer (HR = 6.04, 95% CI 1.82–19.98, p = 0.003) and patients with TNBC (HR = 2.03, 95% CI 1.06–3.88, p = 0.033; Table 2). In addition, an SII cut-off value of ≤ 600 (HR = 1.92, 95% CI 1.20–3.08, p = 0.006) and > 600 (HR = 1.70, 95% CI 1.01–2.88, p = 0.047) predicted poor DFS/RFS. A high SII was associated with poor DFS/RFS of patients receiving mixed treatments (HR = 1.8, 95% CI 1.36–2.42, p < 0.001; Table 2).

### Association between the SII and DMFS of patients with breast cancer

**Fig. 4:**
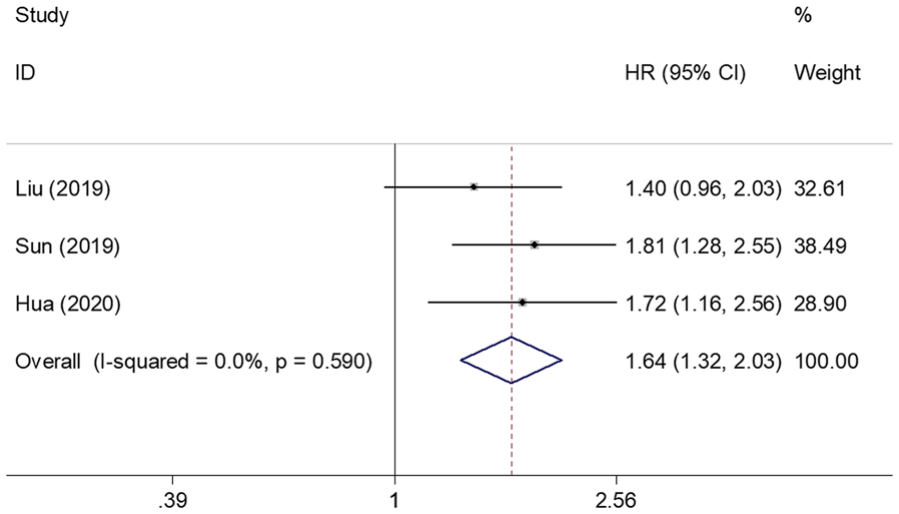
Forest plot of the correlation between SII and distant metastasis-free survival in patients with breast cancer

Three studies involving 1341 patients [11, 12, 16] provided data regarding the prognostic impact of the SII for DMFS. The pooled HR and 95% CI were 1.64 and 1.32–2.03, respectively (p < 0.001), with no significant heterogeneity (*I*^2^ = 0%, p = 0.590; Fig. 4; Table 2). Owing to the limited sample size, subgroup analysis was not performed for DMFS.

### Association between the SII and clinicopathological features of patients with breast cancer

**Fig. 5:**
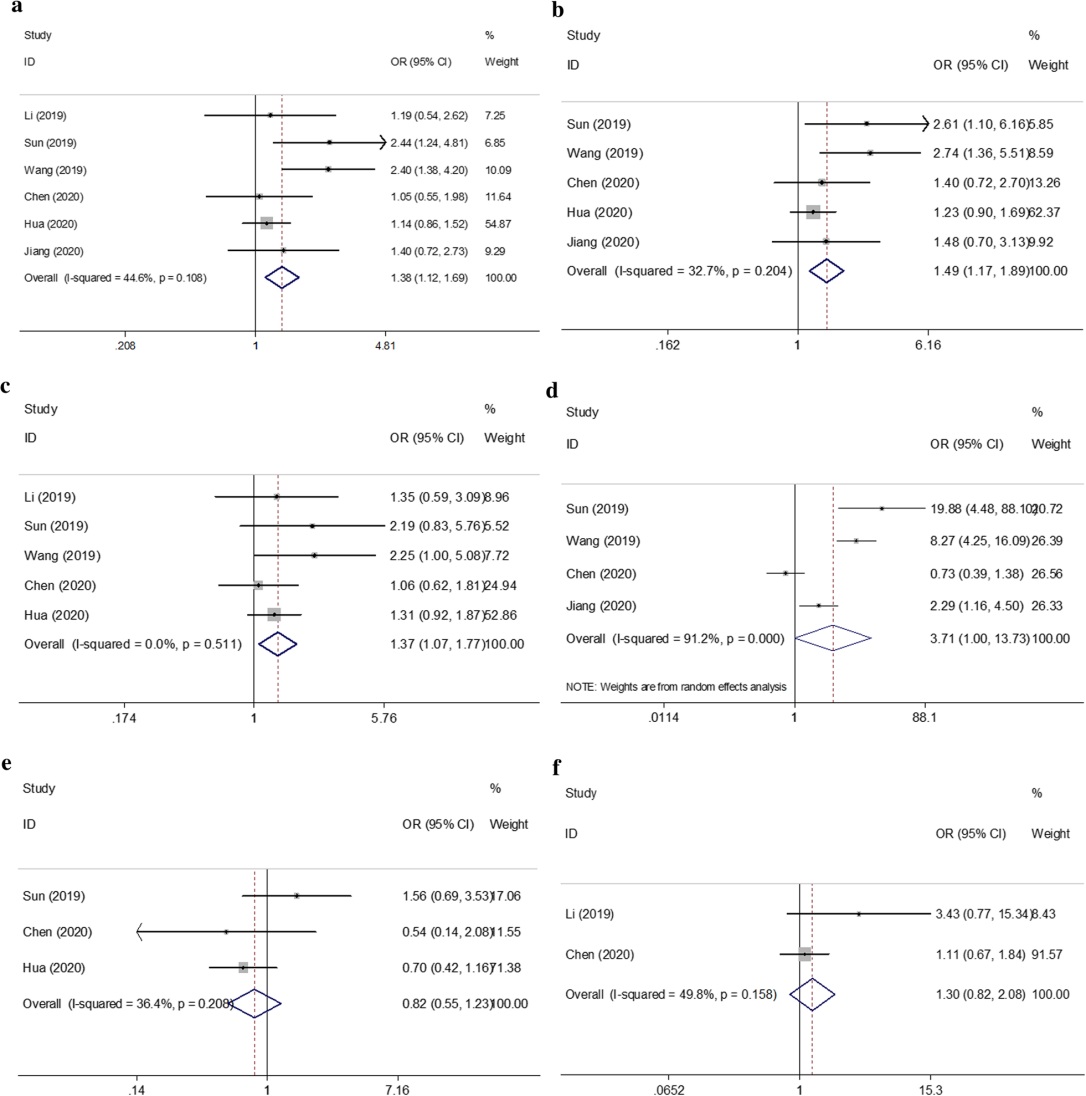
Forest plots for association between SII and various clinicopathological features in breast cancer. **a** presence of lymph node (LN) metastasis (yes vs no); **b** T stage (T2–T4 vs T1); **c** TNM stage (II–III vs 0–I); **d** histological grade (G3 vs G1–G2); **e** pathological type (intralobular carcinoma vs intraductal carcinoma) and **f** lymphatic invasion (yes vs no)

The relationship between the SII and clinicopathological characteristics was analyzed by using data from 6 studies with 1966 patients [10, 12–16]. Six clinicopathological factors were investigated, including lymph node metastasis (presence vs. absence), T stage (T2–T4 vs. T1), TNM stage (II–III vs. 0–I), histological grade (G3 vs. G1–G2), pathological type (intralobular carcinoma vs. intraductal carcinoma), and lymphatic invasion (presence vs. absence). The combined ORs and 95% CIs indicated that a high SII was correlated with the presence of lymph node metastasis (OR = 1.38, 95% CI 1.12–1.69, p = 0.002), higher T stage (OR = 1.49, 95% CI 1.17–1.89, p < 0.001), advanced TNM stage (OR = 1.37, 95% CI 1.07–1.77, p = 0.014), and higher histological grade (OR = 3.71, 95% CI 1.00–13.73, p = 0.049; Fig. 5; Table 3). However, there was no significant association between the SII and pathological type (OR = 0.82, 95% CI 0.55–1.23, p = 0.345) or lymphatic invasion (OR = 1.30, 95% CI 0.82–2.08, p = 0.266; Fig. 5; Table 3).

**Table 3:** Relationship between SII and clinicopathological variables in breast cancer

Clinicopathological features	No. of studies	No. of patients	OR (95% CI)	p	Effects model	Heterogeneity	
						*I*^2^ (%)	p
LN metastasis (yes vs no)	6	1966	1.38 (1.12–1.69)	0.002	Fixed	44.6	0.108
T stage (T2–T4 vs T1)	5	1805	1.49 (1.17–1.89)	0.001	Fixed	32.7	0.204
TNM stage (II–III vs 0–I)	5	1819	1.37 (1.07–1.77)	0.014	Fixed	0	0.511
Histological grade (G3 vs G1–G2)	4	779	3.71 (1.00–13.73)	0.049	Random	91.2	< 0.001
Pathological type (ILC vs IDC)	3	1443	0.82 (0.55–1.23)	0.345	Fixed	36.4	0.208
Lymphatic invasion (yes vs no)	2	423	1.30 (0.82–2.08)	0.266	Fixed	49.8	0.158

*OR* odds ratio, *G* grade, *ILC* intralobular carcinoma, *IDC* intraductal carcinoma

### Publication bias

**Fig. 6:**
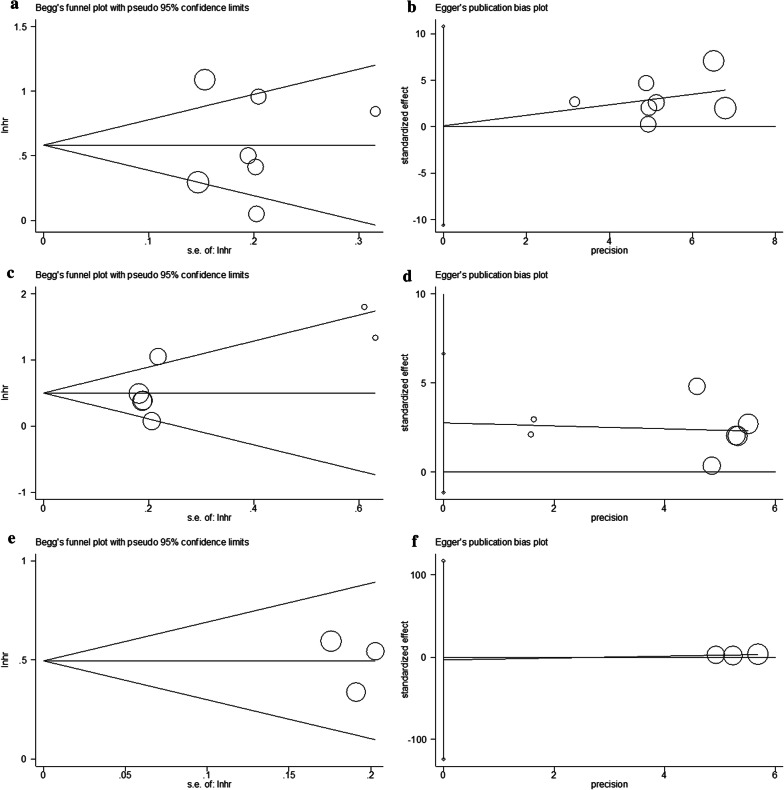
Begg’s funnel plot and Egger’s test for the assessment of publication bias in the meta-analysis. **a** Begg’s test for OS; **b** Egger’s test for OS; **c** Begg’s test for DFS/RFS; **d** Egger’s test for DFS/RFS; **e** Begg’s test for DMFS; and **f** Egger’s test for DMFS

The Begg funnel plot and the Egger test were conducted to evaluate potential publication bias for OS, DFS/RFS, and DMFS analysis. For OS, the test results suggested that the potential publication bias was negative (p = 0.881 on the Begg test, and p = 0.981 on the Egger test; Fig. 6). Similarly, there was no significant publication bias for DFS/RFS (p = 0.548 on the Begg test, and p = 0.128 on the Egger test) or DMFS (p = 0.602 on the Begg test, and p = 0.785 on the Egger test; Fig. 6).

## Discussion

In the current meta-analysis, we evaluated the prognostic influence of the SII in patients with breast cancer. Our results showed that the SII was associated with worse OS, DFS/RFS, and DMFS. Moreover, the prognostic effect of the SII remained consistent for patients with TNBC as well as when the cutoff value of the SII was ≤ 600. We also found that a high SII was associated with the clinical characteristics that indicated tumor progression and high malignancy, including the presence of lymph node metastasis, a higher T stage, advanced TNM stage, and higher histological grade. As the SII is a blood-derived parameter and is easily available, it is an optimal tool for aiding in the prognostication of patients with breast cancer. To the best of our knowledge, the present study is the first meta-analysis to evaluate the prognostic and clinicopathological value of the SII in patients with breast cancer.

The SII is calculated by using the following formula: neutrophil count × platelet count/lymphocyte count; the SII was developed as a prognostic factor for determining the survival outcomes of patients with various cancers in clinical practice [28–34]. As the SII is an index of the combination of neutrophil, platelet, and lymphocyte counts, a high SII could be attributed to the changes in the counts of these cells. Neutrophils can exert tumor-promoting activity by secreting a variety of inflammatory mediators, including vascular endothelial growth factor, interleukin (IL)-6, IL-10, and IL-22 [35]. Platelets can protect cancer cells from lysis by natural killer cells [36] and promote cancer cell arrest in the endothelium, supporting the formation of secondary lesions [37]. In contrast, lymphocytes are involved in cancer immune-surveillance to inhibit cancer progression [38]. Therefore, low lymphocyte counts may result in inadequate immunological reactions in patients with cancer [39].

The prognostic effect of the SII has been studied in many human tumors by using a meta-analysis approach [40–43]. A comprehensive meta-analysis containing 15 articles showed that an SII greater than the cutoff predicted poor OS in various cancers [40]. Moreover, another meta-analysis including 9 studies with 2441 patients revealed that an elevated pretreatment SII indicated significantly poorer OS, DFS/progression-free survival, and cancer-specific survival of patients with non-small cell lung cancer [42]. A recent meta-analysis published in 2020 demonstrated that an elevated SII was a poor prognostic factor for patients with hepatocellular carcinoma [43]. The results of the current meta-analysis extend the prognostic role of the SII for breast cancer. Therefore, we recommend that the SII be used to predict the prognosis of patients with breast cancer.

The current meta-analysis has several limitations. First, significant heterogeneity was detected even though we selected a random-effects model for calculation. Second, most eligible studies were from China; therefore, the results may be more relevant to Chinese patients. The prognostic value of the SII for patients of other nationalities still needs to be verified. Third, the cutoff value of the SII was not uniform among the studies, which may have introduced a selection bias in the meta-analysis.

## Conclusions

In summary, the results of our meta-analysis suggest that an elevated SII predicts poor survival outcomes and is associated with clinicopathological features that indicate tumor progression of breast cancer. However, owing to the several limitations, more prospective studies including patients with diverse ethnicities are needed to confirm our results.

## Data Availability

The datasets analyzed during the current study are available from the corresponding author on reasonable request.

## Abbreviations

SII: Systemic immune-inflammation index
HR: Hazard ratio
CI: Confidence interval
OS: Overall survival
DFS: Disease-free survival
RFS: Recurrence-free survival
DMFS: Distant metastasis-free survival
NLR: Neutrophil–lymphocyte ratio
PLR: Platelet-lymphocyte ratio
CAR: C-reactive protein/albumin ratio
PRISMA: Preferred Reporting Items for Systematic Reviews and Meta-Analyses Statement
NOS: Newcastle–Ottawa quality assessment scale
OR: Odds ratio
TNBC: Triple-negative breast cancer
HER2: Human epidermal growth factor receptor-2
ILC: Intralobular carcinoma
IDC: Intraductal carcinoma
IL-6: Interleukin-6
PFS: Progression-free survival
CSS: Cancer-specific survival
